# Where is the gap?: the contribution of disparities within developing countries to global inequalities in under-five mortality

**DOI:** 10.1186/1471-2458-14-216

**Published:** 2014-03-01

**Authors:** Agbessi Amouzou, Naoko Kozuki, Davidson R Gwatkin

**Affiliations:** 1 Johns Hopkins Bloomberg School of Public Health, 615 N. Wolfe St., 21205 Baltimore, MD, USA; 2 Results for Development Institute, 1100 15th Street NW Suite 400, 20005 Washington, DC, USA

**Keywords:** Under-five mortality, Health inequity

## Abstract

**Background:**

Global health equity strategists have previously focused much on differences across countries. At first glance, the global health gap appears to result primarily from disparities between the developing and developed regions. We examine how much of this disparity could be attributed to within-country disparities in developing nations.

**Methods:**

We used data from Demographic and Health Surveys conducted between 1995 and 2010 in 67 developing countries. Using a population attributable risk approach, we computed the proportion of global under-five mortality gap and the absolute number of under-five deaths that would be reduced if the under-five mortality rate in each of these 67 countries was lowered to the level of the top 10% economic group in each country. As a sensitivity check, we also conducted comparable calculations using top 5% and the top 20% economic group.

**Results:**

In 2007, approximately 6.6 million under-five deaths were observed in the 67 countries used in the analysis. This could be reduced to only 600,000 deaths if these countries had the same under-five mortality rate as developed countries. If the under-five mortality rate was lowered to the rate among the top 10% economic group in each of these countries, under-five deaths would be reduced to 3.7 million. This corresponds to a 48% reduction in the global mortality gap and 2.9 million under-five deaths averted. Using cutoff points of top 5% and top 20% economic groups showed reduction of 37% and 56% respectively in the global mortality gap. With these cutoff points, respectively 2.3 and 3.4 million under-five deaths would be averted.

**Conclusion:**

Under-five mortality disparities within developing countries account for roughly half of the global gap between developed and developing countries. Thus, within-country inequities deserve as much consideration as do inequalities between the world’s developing and developed regions.

## Background

Increased attention has recently been given to economic disparities in health status, health service coverage, and their impact on health improvements in developing countries [1-5]. In these countries, children from poor families have higher risk of death than those from rich families, but paradoxically, rich families are generally the first to take advantage of health interventions that are not aggressively targeted to the poor [1,6,7]. Improvements in maternal, newborn and child health in developing countries require special attention to addressing the health equity gap between poor and rich within and across countries by ensuring that health programs incorporate strategies addressing inequalities [2,3]. Recent studies have demonstrated that countries that have reached high coverage of maternal, newborn, and child health interventions are also those that showed reduced socio-economic inequalities in these coverage levels [4,5,8-10]. Other studies have shown that strategies that focus on equity are cost-effective compared to other mainstream approaches. Several such strategies exist, such as the use of community health workers, outreach campaigns, task shifting, and elimination of user fees [9,10].

On the surface, calls for such a focus on health conditions among the poor within developing countries in order to reduce the global health gap might seem strange. By some standard measures, inequalities between the world’s developed and developing regions are far larger than intra-country disparities. Take the case of under-five mortality rates (U5MR), or the number of children dying between birth and age five per 1000 live births. U5MR is the health status indicator for which the greatest amount of relevant evidence is available and a key indicator for the fourth Millennium Development Goal of reducing U5MR by two-thirds between 1990 and 2015. A recent survey of 56 low- and middle-income countries (LMIC) found that the U5MR within the poorest 20% of a national population during the 1990s was, on average, almost twice as high as that in the population’s best-off 20% [6]. At that time, the average U5MR in the same set of LMICs was over twelve times as high as that in the developed world. Estimates from the UN Interagency Group for Mortality Estimation in 2012 indicate that U5MR in developing regions was about nine times as high as that of the developed regions. In terms of burden of under-five deaths, while about 6.5 million deaths were estimated for developing countries in 2012, only 90,000 deaths were estimated for developed countries [11]. Seen in this light, the disparity within the typical developing country does not seem large enough to merit much concern in the formulation of global health equity strategies, and an emphasis on reducing disparities between developed and developing countries at global level would be justified.

We argue in this paper that such conclusion would be misguided without careful analysis of the magnitude of intra- and inter-country disparities. Such an analysis would examine each kind of disparity’s contribution to the gap between developed and developing countries by applying a variant of the population attributable risk approach, widely used to estimate the reduction in mortality brought about by eliminating a particular cause of death.

The purpose of our study is to determine how much of the under-five mortality gap between developed and developing countries can be reduced by improving mortality rates of developing countries to the level of their highest income groups. Although several studies, such as the “Global Health 2035” Lancet Commission report, [12] have recently drawn attention to health inequities, no known study has attempted to quantify the size of the inequity between developed and developing countries and the relative contribution of the inequality gap within developing countries to the global inequality. Such studies are necessary to provide evidence for health inequality reduction programming.

Our findings highlight the magnitude of mortality burden attributable to within-country inequalities and corroborate the need for focused priority policies toward reducing socioeconomic inequalities in health in developing countries.

## Methods

### Data

To estimate U5MR for different economic strata of developing country populations, we required data that 1) include both information about mortality and socio-economic status, and 2) are large enough to contain information on under-five deaths that provide reliable estimates on subgroups of the population. We selected most recent Demographic and Health Surveys (DHS), which meet the aforementioned criteria. We used the wealth index as indicator of economic status. The wealth index, a composite measure of a household economic status, is derived from household assets and characteristics using principal component analysis [13,14]. The index is widely used to split the household population into economic groups of different levels in order to analyze economic inequalities. Data from before 1994 were excluded due to smaller sample sizes and limited information on asset variables used for the wealth index computation. For countries with multiple surveys, we used only the most recent survey. A total number of 67 DHS datasets were retained (available as of December 2012) and included in the analysis, with a median year of survey of 2007. These 67 DHS countries represent 83.8 million births, or approximately 70% of births in LMICs in 2007. We defined developing countries as those categorized as low- or middle-income countries by the World Development Indicators published by the World Bank (list of countries available in Additional file 1: Table S1) [15].

### Analysis

Data on under-five mortality in the highest economic groups needed for the estimation of the intra-country mortality gap was derived by pooling all country datasets on interviewed women’s birth histories. Prior to pooling those datasets, the wealth index already available in each DHS dataset, was used to split the household population into groups of equal size. The principal focus was on the top 10% (decile) of the sample population; as sensitivity checks, we also selected the top 5% (ventile) and 20% (quintile) of the sampled population. The pooled dataset was used to compute U5MR for the top decile, ventile, and quintile of the total population of the 67 countries, using appropriate adjustment weights. The wealth quintile variable (five subgroups of the household population based on the wealth score) was already available in each country dataset. The pooled U5MR was weighted such that the mortality rate obtained is equivalent to the weighted average of the 67 country-specific U5MRs, with weights representing the proportion of country births in the total number of births. The final weights used in the pooled dataset combined these latter weights with sampling weights provided in each dataset. We computed the U5MR using life table approach with a synthetic cohort approach [16].

Application of the population attributable risk approach to determine the proportion of the total gap that is due to within-country disparities in U5MR involved three steps. The first is estimating the size of the global gap. The second consists of preparing an alternative estimate, to show how large the gap would be if mortality inequalities within developing countries were eliminated in the manner described below – that is, by improving health among low- and middle-income groups to the levels already enjoyed by the highest ones within the countries concerned. The third and final step involves comparing the estimates prepared in steps one and two.

The estimate prepared in the first step will be called the “actual mortality gap” between developed and developing countries. It consists of the observed number of under-five deaths in developing countries, minus the number of such deaths were all economic groups in those countries to enjoy the same U5MR as the average found in developed countries. We calculated the observed number of under-five deaths in developing countries by using country population, crude birth rate, and U5MR in 2007 for all 67 countries included in the analysis. The country population, crude birth rate, and U5MR were taken from the World Development Indicators (Additional file 1: Table S2) [15]. Then using the same data, we calculated the number of under-five deaths that would be observed had each of the 67 countries experienced the U5MR of developed countries in 2007 (7 per 1000 live births) [15]. The actual mortality gap is the difference between the observed under-five deaths and the estimated number of deaths using the developed countries’ U5MR in 2007.

The second estimate will be termed the “reduced mortality gap” between these same two groups of countries. It is the observed number of under-five deaths in developing countries, minus the number of such deaths were the U5MR for each developing country to equal the mortality rate experienced by the country's top economic group. Using the pooled data of the 67 countries, we computed U5MR for the top economic group (top decile, ventile, and quintile) using appropriate weights as described above.

We then applied the U5MR of the top economic group to the total number of births in 2007 in the 67 countries to estimate the number of under-five deaths. The “reduced mortality gap” is the difference between the observed number of under-five deaths in these 67 developing countries in 2007 and the estimated number of deaths using the U5MR in the top economic group in the same 67 developing countries.

In the third step, the estimates produced in the first and second steps are compared. The outcome expressed in relative terms represents the percentage decrease in the actual mortality gap that would result from achieving the reduced gap just described. We used Stata version 12.0 for the analysis.

## Results

The list of 67 DHS datasets used and summary of the data can be found in Additional file 1: Table S2. These data covered approximately 4.2 million representatively-selected individuals (after adjusting for sample weights), and approximately one million births and 90,000 under-five deaths during the ten years prior to each surveys. In these countries, around 83.8 million births and 6.6 million under-five deaths were recorded in 2007, the median year of the DHS surveys included in the analysis. This 6.6 million represents about 72% of the 9.2 million under-five deaths occurring in developing countries that year [17].

The gap analysis results using the top 10% (decile) of the population based on the wealth index as the top economic group are presented in Table 1. It has three sections. Section A represents step one and the estimate produced by it (actual mortality gap). Section B covers the procedure’s second step, and the estimate that it yields (reduced mortality gap). In Section C, the magnitudes of the two are compared.

**Table 1:** Impact of eliminating mortality disparities within developing countries, by lowering mortality rates to those enjoyed by the highest economic decile within those countries: 67 low- and middle-income countries, 2007

***Section A. Actual mortality gap***	
1. Number of births (from Additional file 1: Table S2)	83,849,024
2. Number of under-5 deaths (from Additional file 1: Table S2)	6,555,200
3. No. of deaths at developed countries’ Rate (line 1 × .007)*	586,943
4. Actual mortality gap (line 2 – line 3)	5,968,257
***Section B. Reduced mortality gap***	
5. Number of births (from Additional file 1: Table S2)	83,849,024
6. Number of under-5 deaths (from Additional file 1: Table S2)	6,555,200
7. No. of deaths at rate of countries’ best-off group (line 5 × top decile rate of 0.0441 deaths per live birth**)	3,697,742
8. Reduced mortality gap (line 6 – line 7)	2,857,458
***Section C. Reduction in actual mortality gap by achieving reduced mortality gap***	
9. Reduced mortality gap (from line 8)	2,857,458
10. Actual mortality gap (from line 4)	5,968,257
11. Percent reduction in actual gap by achieving reduced gap (line 9 ÷ line 10)	47.9%

*The World Bank, World Development Indicators, 2012 (Washington, D.C.: The World Bank, 2012).

http://data.worldbank.org/data-catalog/world-development-indicators.

**The mortality rate was not rounded for the calculation, so the value Line 7 differs slightly from Line 5 × 0.0441 (the rounded mortality rate).

Lines 1 and 2 of Table 1, Section A are taken from Additional file 1: Table S2; they show the total number of births and under-five deaths in the 67 countries covered. Line 3 shows the number of under-five deaths that would have occurred in these 67 countries had the births on line 1 been subject to the U5MR of 7 per 1,000 live births that prevailed in developed countries in 2007. The difference between lines 2 and 3, which appears on line 4, represents the number of under-five deaths that would have been averted in the 67 countries covered if their U5MR had been that of the developed countries rather than their actual U5MR.

In Section B, the same analysis is repeated, only now using the U5MR of 44.1 deaths per 1000 live births, the mortality rate experienced in the top economic decile of the included 67 developing countries. Line 7 represents the number of under-five deaths that would have occurred in the 67 countries had their average U5MR been 44.1 deaths per 1000 live births. (The U5MR of each wealth decile, as well as of each wealth ventile and quintile, appears in Additional file 1: Table S3a-c.). The reduced mortality gap is the difference between line six and line seven, and is reported on line eight.

The third and final step of the procedure, shown in Section C, produces the outcome of interest. This outcome comes from dividing the size of the reduced gap (2,857,458, on line 9) by the actual gap (5,968,257 on line 10). The result is 48%, indicating that the actual global mortality gap could be reduced by 48% if the U5MR in all economic groups in developing country populations could be lowered to that of the top 10% in those populations. As can be seen from line 8 of the table, this would result in around 2.9 million fewer under-five deaths.

The analyses using mortality rates from the top wealth quintile and ventile are available in Additional file 1: Table S4a and b. The mortality gap would be reduced by 38% and 57% respectively.

## Discussion

Using a population attributable risk approach, we demonstrated that about half of the under-five mortality gap between developed and developing countries can be reduced by lowering U5MR in each developing country to the level enjoyed by the top economic stratum of these countries. For our purposes, these developing countries were represented by the 67 countries with available DHS data. An accurate appreciation of the findings’ implications requires a clear awareness of the many nuances in the approach taken and the data used. In the present case, three of the nuances are especially noteworthy.

First, the size of the intra-country mortality gap inevitably depends upon how the best-off group is defined, and there is no known conceptually-satisfying definition for such a group. For example, it is likely that there exists within even the poorest countries some very small but highly privileged groups with mortality rates close to or even better than those of the industrialized world. Were this small group to be taken as the best-off for the purposes of the calculations performed here, then almost all of the global mortality gap would be attributable to differences within countries. Using lower economic cut-offs (e.g. everyone above the poverty line) would produce a higher U5MR among the reference population we are using as “best off,” and a correspondingly lower portion of the global gap resulting from intra-country disparities.

While the top wealth decile that we used in our analysis has served as the indicator of choice whenever the available datasets are adequately large, it is largely arbitrary. Plausible alternatives exist, and their use could produce notably different results. For example, using the top 5% of the population as the reference group would increase the relative difference between the two gap measures from 48% to 57%, and the absolute difference from 2.9 million to 3.4 million under-five deaths. Had the reference group been the population’s top 20%, the gap reported would have fallen to 38% and 2.3 million under-five deaths. Such variability argues for considering the results produced based on a 10% figure as no more than a general sense of the magnitudes involved. It would be safer to say, for example, that eliminating U5MR disparities within developing countries by reducing U5MR among developing countries could reasonably be expected to reduce the actual global mortality gap by roughly a half, and avert two to three million under-five deaths.

Second, while the population attributable risk approach used here is quite standard, it also represents no more than a very schematic approximation of reality. In actuality, it is obviously unrealistic to expect a sudden decline in under-five mortality that would leave other important parameters unchanged. Far more likely would be an extended process of change, involving a wide range of broad social and economic developments, as well as health service modifications, shifting the relative economic positions of different groups, and influencing fertility as well as mortality. Whether such a process might lead to results similar to those reported here is difficult to say. The analysis presented does not however make any assumption or claims about the determinants or causes of mortality change.

Finally, the DHS data used were collected between 1994 and 2011 and the mortality rates computed referred to a period ten years preceding each survey. The range in survey years introduces variability in the data. It should be noted that our analysis utilizes estimates that are averages over this period.

Findings in our study suggest that the global under-five mortality gap could be reduced by roughly half by eliminating intra-country U5MR disparities by economic status, and doing so could avert around two to three million deaths annually. The findings presented cannot plausibly be viewed as providing more than general orders of magnitude. However, our findings support two principal points: that economic inequalities with respect to health within developing countries contribute much more to the global health gap than might appear to be the case at first glance and that these inequalities deserve at least as much attention as does the gap between those countries and developed countries.

## Conclusion

Over six million more under-five deaths occur in developing countries than in developed countries every year. Under-five mortality disparities within developing countries account for roughly half of that mortality gap. Within-country inequities require as much consideration as do inequities between the world’s developing and developed regions.

## Competing interests

The authors report no competing interests.

## Authors’ contributions

AA and DRG designed the study, AA, NK, and DRG analyzed the data, AA, NK, and DRG contributed to the writing of the manuscript. All authors read and approved the final manuscript.

## Pre-publication history

The pre-publication history for this paper can be accessed here:

http://www.biomedcentral.com/1471-2458/14/216/prepub

## Supplementary Material

Additional file 1: Table S1 List of countries by World Bank income categories. **Table S2.** Demographic and Health Survey and population data from 67 low- and middle-income countries contributing to the analysis. **Table S3a-c.** Pooled U5MR by wealth quintiles, deciles, and ventiles for 67 developing countries included in the analysis. **Table S4a.** Impact of eliminating mortality disparities within developing countries, by lowering mortality rates to those enjoyed by the highest wealth quintile within those countries: 67 Low- and Middle-Income Countries, 2007. **Table S4b.** Impact of eliminating mortality disparities within developing countries, by lowering mortality rates to those enjoyed by the highest wealth ventile within those countries: 67 Low- and Middle-Income Countries, 2007. Click here for file
