# Supplementary material for: Where is the gap?: the contribution of disparities within developing countries to global inequalities in under-five mortality
Source: BMC Public Health. 2014 Mar 1;14:216. doi: 10.1186/1471-2458-14-216 (PMC3945794; doi:10.1186/1471-2458-14-216)
Supplement: Additional file 1: Table S1 — List of countries by World Bank income categories. Table S2. Demographic and Health Survey and population data from 67 low- and middle-income countries contributing to the analysis. Table S3a-c. Pooled U5MR by wealth quintiles, deciles, and ventiles for 67 developing countries included in the analysis. Table S4a. Impact of eliminating mortality disparities within developing countries, by lowering mortality rates to those enjoyed by the highest wealth quintile within those countries: 67 Low- and Middle-Income Countries, 2007. Table S4b. Impact of eliminating mortality disparities within developing countries, by lowering mortality rates to those enjoyed by the highest wealth ventile within those countries: 67 Low- and Middle-Income Countries, 2007. [file 1471-2458-14-216-S1.docx]

**Supplemental Materials**

**Table S1: List of countries by World Bank income categories**

| Low income | Afghanistan, Burundi, Benin, Burkina Faso, Bangladesh, Central African Republic, Comoros, Eritrea, Ethiopia, Guinea, The Gambia, Guinea-Bissau, Haiti, Kenya, Kyrgyz Republic, Cambodia, Liberia, Madagascar, Mali, Myanmar, Mozambique, Malawi, Niger, Nepal, Democratic People's Republic of Korea, Rwanda, Sierra Leone, Somalia, South Sudan, Chad, Togo, Tajikistan, Tanzania, Uganda, Democratic Republic of Congo, Zimbabwe |
| --- | --- |
| Lower middle income | Armenia, Bolivia, Bhutan, Cote d'Ivoire, Cameroon, Congo, Cape Verde, Djibouti, Egypt, Micronesia, Georgia, Ghana, Guatemala, Guyana, Honduras, Indonesia, India, Kiribati, Kosovo, Lao PDR, Sri Lanka, Lesotho, Morocco, Moldova, Mongolia, Mauritania, Nigeria, Nicaragua, Pakistan, Philippines, Papua New Guinea, Paraguay, Sudan, Senegal, Solomon Islands, El Salvador, Sao Tome and Principe, Swaziland, Syrian Arab republic, Timor-Leste, Ukraine, Uzbekistan, Vietnam, Vanuatu, West Bank and Gaza, Samoa, Yemen, Zambia |
| Upper middle income | Angola, Albania, Argentina, American Samoa, Azerbaijan, Bulgaria, Bosnia and Herzegovina, Belarus, Belize, Brazil, Botswana, China, Colombia, Costa Rica, Cuba, Dominica, Dominican Republic, Algeria, Ecuador, Fiji, Gabon, Grenada, Hungary, Iran, Iraq, Jamaica, Jordan, Kazakhstan, Lebanon, Libya, St. Lucia, Maldives, Mexico, Marshall Islands, Macedonia, Montenegro, Mauritius, Malaysia, Namibia, Panama, Peru, Palau, Romania, Serbia, Surname, Seychelles, Thailand, Turkmenistan, Tongo, Tunisia, Turkey, Tuvalu, St. Vincent and the Grenadines, Venezuela, South Africa |
| High income: nonOECD | Aruba, Andorra, United Arab Emirates, Antigua and Barbuda, Bahrain, The Bahamas, Bermuda, Barbados, Brunei, Channel Islands, Curacao, Cayman Islands, Cyprus, Faeroe Islands, Equatorial Guinea, Greenland, Guam, Hong Kong SAR (China), Croatia, Isle of Man, St. Kitts and Nevis, Kuwait, Liechtenstein, Lithuania, Latvia, Macao SAR (China), St. Martin (French), Monaco, Malta, Northern Mariana Islands, New Caledonia, Oman, Puerto Rico, French Polynesia, Qatar Russia, Saudi Arabia, Singapore, San Marino, Sint Maarten (Dutch), Turks and Caicos Islands, Trinidad and Tobago, Uruguay, Virgin Islands |
| High income: OECD | Australia, Austria, Belgium, Canada, Switzerland, Chile, Czech Republic, Germany, Denmark, Spain, Estonia, Finland, France, United Kingdom, Greece, Ireland, Iceland, Israel, Italy, Japan, Korea, Luxembourg, Netherlands, Norway, New Zealand, Poland, Portugal, Slovak Republic, Slovenia, Sweden, United States |

Source: [[15](#_ENREF_15)]

**Table S2: Demographic and Health Survey and population data from 67 low- and middle-income countries contributing to the analysis**

| **Country and year of survey** | **Demographic and Health Survey data** | | | **Population data** | | | | | |
| --- | --- | --- | --- | --- | --- | --- | --- | --- | --- |
|  | **Individuals covered** | **Number of births recorded*** | **Ten-year U5MR from DHS survey** | **Total Population in year of survey**** | **Total population in 2007**** | **Crude birth rate in 2007**** | **Average births in 2007**** | **U5MR in 2007**** | **Average under-five deaths in 2007** |
| Albania 2008 | 30,521 | 3,925 | 22 | 3,181,397 | 3169665 | 13.0 | 41336 | 17.8 | 736 |
| Armenia 2010 | 23,787 | 2,803 | 21 | 3,092,072 | 3074181 | 15.1 | 46497 | 21.1 | 981 |
| Azerbaijan 2006 | 29,674 | 4,564 | 58 | 8,484,550 | 8581300 | 18.0 | 154463 | 52.9 | 8171 |
| Bangladesh 2007 | 48,919 | 12,615 | 74 | 143,956,805 | 143956805 | 21.9 | 3151934 | 57.4 | 180921 |
| Benin 2006 | 88,316 | 30,551 | 136 | 7,871,707 | 8112573 | 40.8 | 331366 | 117.2 | 38836 |
| Bolivia 2008 | 77,081 | 18,436 | 76 | 9,618,466 | 9463497 | 27.7 | 262167 | 59.8 | 15678 |
| Brazil 1996 | 54,232 | 10,357 | 57 | 163,819,245 | 189798070 | 16.8 | 3186330 | 21.2 | 67550 |
| Burkina Faso 2010 | 81,831 | 30,131 | 148 | 16,468,714 | 15061127 | 44.0 | 662223 | 157.3 | 104168 |
| Burundi 2010 | 41,145 | 14,473 | 127 | 8,382,849 | 7707781 | 34.4 | 265441 | 148.1 | 39312 |
| Cambodia 2010 | 74,416 | 16,116 | 68 | 14,138,255 | 13669857 | 23.3 | 318644 | 58.0 | 18481 |
| Cameroon 2011 | 70,882 | 22,264 | 128 | 20,030,362 | 18350022 | 37.3 | 685025 | 133.5 | 91451 |
| Central African Republic 1994-95 | 27,168 | 9,367 | 159 | 3,331,355 | 4160940 | 35.9 | 149548 | 167.6 | 25064 |
| Chad 2004 | 28,747 | 12,018 | 203 | 9,474,792 | 10371839 | 46.2 | 478982 | 176.1 | 84349 |
| Colombia 2010 | 194,004 | 33,009 | 22 | 46,294,841 | 44352327 | 20.7 | 917783 | 20.0 | 18356 |
| Comoros 1996 | 14,188 | 4,025 | 113 | 496,567 | 678602 | 39.2 | 26628 | 87.0 | 2317 |
| Congo Republic 2005 | 30,337 | 8,916 | 123 | 3,533,177 | 3730612 | 36.2 | 134899 | 102.1 | 13773 |
| Cote D'Ivoire 1998 | 13,079 | 4,378 | 174 | 15,878,555 | 18646754 | 35.2 | 657037 | 123.8 | 81341 |
| Democratic Republic of Congo 2007 | 48,381 | 16,431 | 155 | 60,772,175 | 60772175 | 45.4 | 2756018 | 176.7 | 486988 |
| Dominican Republic 2007 | 120,031 | 22,574 | 37 | 9,531,954 | 9531954 | 22.8 | 217729 | 29.0 | 6314 |
| Egypt 2008 | 87,480 | 21,161 | 33 | 78,323,298 | 76941572 | 24.0 | 1847675 | 27.6 | 50996 |
| Ethiopia 2011 | 77,377 | 24,093 | 110 | 84,734,262 | 77718436 | 33.9 | 2633023 | 95.8 | 252244 |
| Gabon 2000 | 30,733 | 7,775 | 91 | 1,272,094 | 1423637 | 27.6 | 39314 | 73.2 | 2878 |
| Ghana 2007 | 44,080 | 5,647 | 85 | 22,712,403 | 22712403 | 32.7 | 743241 | 84.6 | 62878 |
| Guatemala 1995 | 59,247 | 8,567 | 45 | 10,664,741 | 13358842 | 33.6 | 448349 | 36.1 | 16185 |
| Guinea 2005 | 38,245 | 13,427 | 188 | 9,041,448 | 9373619 | 40.1 | 375498 | 141.6 | 53170 |
| Guyana 2009 | 21,317 | 3,993 | 39 | 753,013 | 750068 | 18.9 | 14143 | 40.3 | 570 |
| Haiti 2005 | 46,055 | 11,462 | 102 | 9,347,262 | 9608453 | 27.7 | 266096 | 80.0 | 21288 |
| Honduras 2007 | 89,662 | 21,783 | 37 | 7,158,819 | 7158819 | 27.9 | 199595 | 25.6 | 5110 |
| India 2005 | 522,027 | 119,960 | 85 | 1,140,042,863 | 1173971629 | 23.2 | 27226750 | 70.0 | 1905873 |
| Indonesia 2007 | 167,002 | 33,256 | 51 | 232,461,746 | 232461746 | 19.4 | 4500692 | 38.2 | 171926 |
| Jordan 2007 | 76,681 | 19,064 | 31 | 5,662,000 | 5662000 | 26.4 | 149460 | 23.2 | 3467 |
| Kazakhstan 1999 | 21,029 | 3,472 | 63 | 14,928,426 | 15484192 | 20.8 | 321916 | 32.8 | 10559 |
| Kenya 2008 | 38,069 | 11,451 | 84 | 38,455,418 | 37485246 | 38.2 | 1431786 | 86.5 | 123850 |
| Kyrgyzstan 1997 | 17,074 | 4,181 | 76 | 4,725,000 | 5268400 | 23.5 | 123807 | 35.8 | 4432 |
| Lesotho 2009 | 33,561 | 6,780 | 105 | 2,149,201 | 2106128 | 28.7 | 60448 | 110.7 | 6692 |
| Liberia 2007 | 34,326 | 10,841 | 158 | 3,477,197 | 3477197 | 40.7 | 141630 | 103.6 | 14673 |
| Madagascar 2008 | 84,333 | 25,691 | 82 | 19,546,282 | 18980391 | 36.4 | 690810 | 74.2 | 51258 |
| Malawi 2010 | 115,100 | 38,184 | 127 | 14,900,841 | 13589404 | 43.8 | 595406 | 109.7 | 65316 |
| Maldives 2009 | 40,980 | 7,054 | 27 | 311,739 | 303539 | 17.3 | 5263 | 19.3 | 102 |
| Mali 2006 | 73,848 | 28,222 | 215 | 13,592,796 | 14020786 | 47.8 | 670684 | 188.8 | 126625 |
| Moldova 2005 | 31,205 | 3,215 | 26 | 3,595,182 | 3576904 | 12.1 | 43352 | 18.5 | 802 |
| Morocco 2003 | 61,635 | 12,594 | 54 | 28,813,986 | 31011199 | 20.2 | 625093 | 39.1 | 24441 |
| Mozambique 2003 | 59,768 | 20,581 | 178 | 19,721,009 | 21811326 | 39.9 | 871013 | 125.9 | 109661 |
| Namibia 2006 | 41,247 | 9,572 | 69 | 2,118,653 | 2158984 | 27.6 | 59648 | 58.6 | 3495 |
| Nepal 2011 | 48,123 | 11,273 | 62 | 30,485,798 | 28373838 | 26.2 | 742345 | 58.4 | 43353 |
| Nicaragua 2001 | 59,898 | 13,974 | 41 | 4,991,475 | 5563654 | 24.8 | 138118 | 30.9 | 4268 |
| Niger 2006 | 46,692 | 20,347 | 218 | 13,460,138 | 13945662 | 49.6 | 692137 | 152.3 | 105412 |
| Nigeria 2008 | 150,589 | 53,858 | 171 | 150,665,730 | 146951477 | 40.5 | 5957707 | 144.6 | 861484 |
| Pakistan 2006 | 66,145 | 18,753 | 93 | 161,513,324 | 164445596 | 27.9 | 4593623 | 80.0 | 367490 |
| Peru 2000 | 130,237 | 25,848 | 33 | 25,952,191 | 28166078 | 21.4 | 602895 | 23.9 | 14409 |
| Philippines 2008 | 59,617 | 12,887 | 37 | 90,173,139 | 88652631 | 26.3 | 2334578 | 29.5 | 68870 |
| Rwanda 2010 | 55,585 | 17,755 | 102 | 10,624,005 | 9710531 | 40.6 | 394636 | 86.0 | 33939 |
| Sao Tome and Principe 2008 | 13,552 | 3,471 | 72 | 159,852 | 157361 | 32.7 | 5146 | 89.6 | 461 |
| Senegal 2010 | 73,645 | 21,495 | 87 | 12,433,728 | 11474661 | 38.7 | 444264 | 84.7 | 37629 |
| Sierra Leone 2008 | 42,805 | 11,994 | 168 | 5,612,129 | 5478289 | 40.8 | 223443 | 204.0 | 45582 |
| South Africa 1998 | 52,007 | 10,376 | 57 | 41,899,683 | 48257282 | 22.2 | 1070877 | 71.1 | 76139 |
| Swaziland 2006 | 22,303 | 5,367 | 106 | 1,018,758 | 1020102 | 30.2 | 30827 | 120.6 | 3718 |
| Tanzania 2010 | 47,728 | 15,352 | 92 | 34,037,979 | 41068185 | 41.7 | 1710531 | 87.2 | 149158 |
| Timor-Leste 2009 | 66,985 | 19,599 | 80 | 1,110,071 | 1046030 | 39.2 | 41027 | 70.0 | 2872 |
| Togo 1998 | 40,388 | 13,414 | 144 | 5,011,183 | 5652811 | 33.5 | 189200 | 116.5 | 22042 |
| Turkey 2003 | 43,977 | 8,525 | 60 | 66,339,433 | 69992754 | 18.7 | 1306905 | 20.6 | 26922 |
| Uganda 2011 | 44,250 | 15,487 | 106 | 34,509,205 | 30339895 | 46.5 | 1412049 | 106.6 | 150524 |
| Ukraine 2007 | 32,809 | 2,443 | 19 | 46,509,350 | 46509350 | 10.2 | 474395 | 12.7 | 6025 |
| Uzbekistan 1996 | 19,388 | 4,957 | 55 | 23,225,000 | 26868000 | 22.7 | 608917 | 52.9 | 32212 |
| Vietnam 2002 | 31,035 | 5,355 | 33 | 80,423,993 | 84221100 | 17.1 | 1442455 | 25.6 | 36927 |
| Zambia 2007 | 34,980 | 11,830 | 137 | 12,055,384 | 12055384 | 44.6 | 537393 | 113.4 | 60940 |
| Zimbabwe 2010 | 40,401 | 10,097 | 77 | 12,571,454 | 12481245 | 29.4 | 366811 | 86.0 | 31546 |
| **All countries** | **4,231,870** | **1,083,433** |  | **3,181,650,519** | **3,256,036,917** |  | **83,849,024** |  | **6,555,200** |

* During the ten years preceding the surveys

** Source: [[15](#_ENREF_15)]. 2007 is the median year of births covered by the country surveys used.

**Table S3a-c:** Pooled under-five mortality rate (U5MR) by wealth quintiles, deciles, and ventiles for 67 developing countries included in the analysis

**3a**

| Wealth quintiles | U5MR |
| --- | --- |
| Quintile 1 | 115.8 |
| Quintile 2 | 101.6 |
| Quintile 3 | 86.5 |
| Quintile 4 | 71.9 |
| Quintile 5 | 51.3 |
| Total | 89.1 |

**3b**

| Wealth deciles | U5MR |
| --- | --- |
| Decile 1 | 118.9 |
| Decile 2 | 113.2 |
| Decile 3 | 104.0 |
| Decile 4 | 98.2 |
| Decile 5 | 90.7 |
| Decile 6 | 81.8 |
| Decile 7 | 75.4 |
| Decile 8 | 68.1 |
| Decile 9 | 57.3 |
| Decile 10 | 44.1 |
| Total | 89.1 |

**3c**

| Wealth ventiles | U5MR |
| --- | --- |
| Ventile 1 | 121.2 |
| Ventile 2 | 116.4 |
| Ventile 3 | 113.4 |
| Ventile 4 | 112.9 |
| Ventile 5 | 104.6 |
| Ventile 6 | 103.3 |
| Ventile 7 | 99.8 |
| Ventile 8 | 96.6 |
| Ventile 9 | 91.3 |
| Ventile 10 | 90.1 |
| Ventile 11 | 81.6 |
| Ventile 12 | 82.1 |
| Ventile 13 | 74.42 |
| Ventile 14 | 76.3 |
| Ventile 15 | 66.8 |
| Ventile 16 | 69.4 |
| Ventile 17 | 61.1 |
| Ventile 18 | 53.3 |
| Ventile 19 | 49.5 |
| Ventile 20 | 37.8 |
| Total | 89.1 |

*Highest quintile/decile/ventile numbers (quintile 5, decile 10, ventile 20) represent the wealthiest stratum.

**Table S4a.** Impact of eliminating mortality disparities within developing countries, by lowering mortality rates to those enjoyed by the highest wealth **quintile** within those countries: 67 Low- and Middle-Income Countries, 2007

| ***Section A. Actual Mortality Gap*** | |
| --- | --- |
| 1. Number of Births (from Table 1) | 83,849,024 |
| 2. Number of Under-5 Deaths (from  Table 1) | 6,555,200 |
| 3. No. of Deaths at developed countries’ Rate (line 1 x .007)* | 586,943 |
| 4. Actual Mortality Gap  (line 2 – line 3) | 5,968,257 |
| ***Section B. Reduced Mortality Gap*** | |
| 5. Number of Births (from Table 1) | 83,849,024 |
| 6. Number of Under-5 Deaths (from  Table 1) | 6,555,200 |
| 7. No. of Deaths at Rate of Countries’ Best-Off Group (line 5 x top **quintile** mortality rate of 0.0513 deaths per live birth**) | 4,300,365 |
| 8. Reduced Mortality Gap  (line 6 – line 7) | 2,254,835 |
| ***Section C; Reduction in Actual Mortality Gap by Achieving Reduced Mortality Gap*** | |
| 9. Reduced Mortality Gap  (from line 8) | 2,243,835 |
| 10. Actual Mortality Gap  (from line 4) | 5,968,257 |
| 11. % Reduction in Actual Gap by  Achieving Reduced Gap  (line 9 ÷ line 10) | 37.8% |

*Source: [[15](#_ENREF_15)]

**The mortality rate was not rounded for the calculation, so the value Line 7 differs slightly from Line 5 x 0.0513 (the rounded mortality rate).

**Table S4b.** Impact of eliminating mortality disparities within developing countries, by lowering mortality rates to those enjoyed by the highest wealth **ventile** within those countries: 67 Low- and Middle-Income Countries, 2007

| ***Section A. Actual Mortality Gap*** | |
| --- | --- |
| 1. Number of Births (from Table 1) | 83,849,024 |
| 2. Number of Under-5 Deaths (from  Table 1) | 6,555,200 |
| 3. No. of Deaths at developed countries’ Rate (line 1 x .007)* | 586,943 |
| 4. Actual Mortality Gap  (line 2 – line 3) | 5,968,257 |
| ***Section B. Reduced Mortality Gap*** | |
| 5. Number of Births (from Table 1) | 83,849,024 |
| 6. Number of Under-5 Deaths (from  Table 1) | 6,555,200 |
| 7. No. of Deaths at Rate of Countries’ Best-Off Group (line 5 x top **ventile** rate of 0.0378 deaths per live birth**) | 3,170,583 |
| 8. Reduced Mortality Gap  (line 6 – line 7) | 3,384,617 |
| ***Section C; Reduction in Actual Mortality Gap by Achieving Reduced Mortality Gap*** | |
| 9. Reduced Mortality Gap  (from line 8) | 3,384,617 |
| 10. Actual Mortality Gap  (from line 4) | 5,968,257 |
| 11. % Reduction in Actual Gap by  Achieving Reduced Gap  (line 9 ÷ line 10) | 56.7% |

*Source: [[15](#_ENREF_15)]

**The mortality rate was not rounded for the calculation, so the value Line 7 differs slightly from Line 5 x 0.0378 (the rounded mortality rate).
